# Tufted Angioma in Children: Report of Two Cases and a Review of the Literature

**DOI:** 10.1155/2014/942489

**Published:** 2014-11-04

**Authors:** Alessandra Dutra da Silva, Grasieli de Oliveira Ramos, Rita Fabiane Teixeira Gomes, Marco Antônio Trevizani Martins, Marcelo Lazzaron Lamers, Manoel Santa'Ana Filho, Pantelis Varvaki Rados, Laura de Campos Hildebrand, Fernanda Visioli

**Affiliations:** ^1^Department of Oral Pathology, Federal University of Rio Grande do Sul, Room 503, Rua Ramiro Barcelos 2492, 90035-003 Porto Alegre, RS, Brazil; ^2^Department of Morphologic Science, Federal University of Rio Grande do Sul, Rua Sarmento Leite 500, 90050-170 Porto Alegre, RS, Brazil

## Abstract

Tufted angioma (TA) is a benign vascular tumor with endothelial origin. It is extremely rare in oral mucosa; only seven cases have been reported in the literature so far. Here, we describe two cases of tufted angioma observed in children and we also present a review of the literature about this pathology, concerning the differential diagnosis and management of this lesion in children.

## 1. Introduction 

Tufted angioma (TA) is a rare benign vascular tumor with endothelial origin characterized by slow and indolent growths [1–3]. This lesion was first described by Nakagawa in 1949 [4], who named it as angioblastoma and, later, Macmillan and Champion [5] renamed it as progressive capillary haemangioma. In 1989, Jones and Orkin defined the term tufted angioma based on the microscopic features of this pathology because the endothelial cell proliferation presents a typical organization in tufts [6].

The TA most common form of presentation is an erythematous macula or plaque with angiomatous appearance [3, 7, 8]. Its localization is typically on the skin of the neck, thorax, shoulders, and extremities [1, 2, 7, 9], and it commonly affects children and young adults [1, 3]. Mucosa involvement is rare [1, 10]; the first case affecting oral mucosa was described by Kleinegger et al. in 2000 [11].

The aim of this paper is to report two cases of tufted angioma affecting children and to review the literature about this pathology, discussing the differential diagnosis and management of these lesions. A bibliographic search was performed up to October of 2014 on online databases (PubMed, Lilacs, Bireme, and ISI), using the key words “tufted angioma,” “oral tufted angioma,” “oral angioma,” “lip OR tongue OR buccal mucosa OR oral mucosa OR mouth AND tufted angioma.” The literature search retrieved 6 papers that reported a total of 7 cases. The clinical features of the oral mucosa cases are reviewed in Table 1.

## 2. Case  1

A 10-year-old male presented to the Center of Dental Specialties of the Federal University of Rio Grande do Sul, Porto Alegre, Brazil, with a lesion localized in the left labial commissure of two months of duration. The lesion was asymptomatic and has rapidly grown in size since it was first noticed.

**Figure 1:**
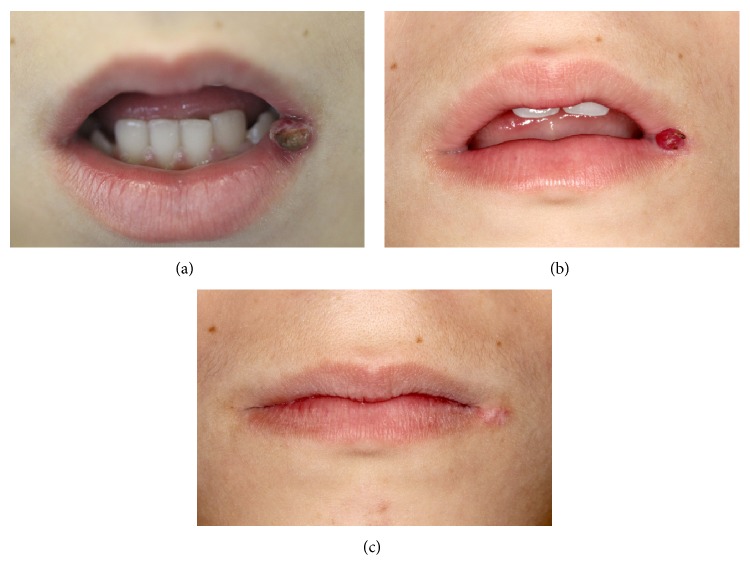
Clinical presentation of case 1. (a) Initial presentation shows a red to purple nodule with a sessile base, measuring approximately 1 cm in diameter. (b) Recurrence of the lesion in the same anatomic site. (c) One-year follow-up clinical appearance.

The clinical examination revealed a firm, red to purple nodule with a sessile base, measuring approximately 1 cm in diameter. The lesion was diagnosed clinically as pyogenic granuloma due to its clinical appearance and because patient reported trauma in the region before the appearance of the lesion (Figure 1(a)).

**Figure 2:**
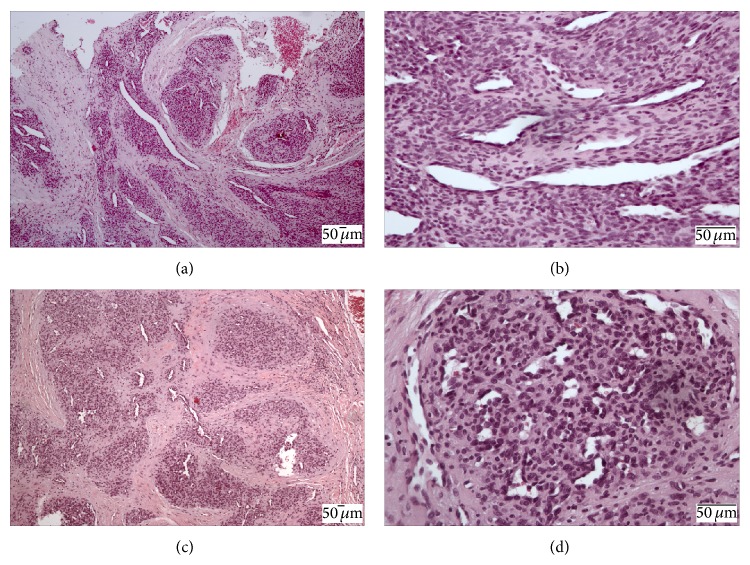
The microscopic examination revealed an endothelial cell proliferation in nodular arrangements with a “cannon ball” pattern covered by oral mucosa. The formation of irregular capillary blood vessels with a slit-shaped lumen was also observed. (a) and (b): microscopic features of case 1. (c) and (d): microscopic features of case 2.

Based on the clinical diagnosis of pyogenic granuloma an excisional biopsy was performed. The microscopic examination revealed an endothelial cell proliferation in nodular arrangements with a “cannon ball” pattern covered by oral mucosa. The formation of irregular capillary blood vessels with a slit-shaped lumen and presence of discrete inflammatory infiltrate in the underlying connective tissue were also observed. Based on the histological features of the lesion, the final diagnosis was tufted angioma (Figures 2(a) and 2(b)).

After 2 weeks, patient returned with recurrence of the lesion in the same anatomic site and reported trauma in the wound area. The clinical examination showed a red nodule with 0.5 cm in diameter (Figure 1(b)). The recurrent lesion was completely excised. No variation of the microscopic characteristics was observed; therefore, the diagnosis of tufted angioma was confirmed. The patient continues to be clinically monitored, without recurrence, after 1 year of follow-up (Figure 1(c)).

## 3. Case  2

**Figure 3:**
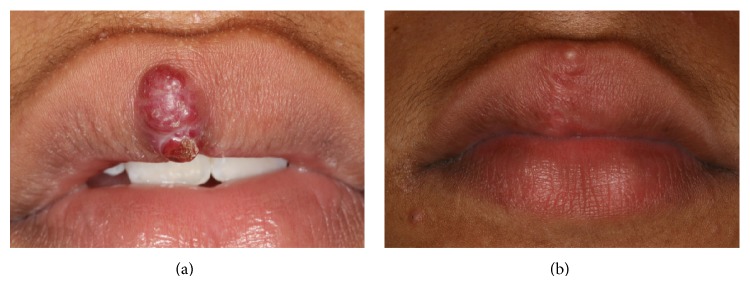
Clinical presentation of case 2. (a) Initial presentation revealed a firm asymptomatic nodule with reddish color, measuring approximately 1 cm in diameter. (b) Presentation at 6-month follow-up.

A 12-year-old male presented to the Center of Dental Specialties of the Federal University of Rio Grande do Sul, Porto Alegre, Brazil, with a nodule localized in upper lip with one month of duration. The patient related a previous lesion with 4-month-period lesion at the same region, which was removed in another service; however no histopathological analysis was performed. After one month of the procedure, the lesion relapsed, and the patient was referred to our service. The clinical examination revealed a firm nodule with reddish color, being asymptomatic, measuring approximately 1 cm in diameter (Figure 3(a)). The lesion was diagnosed clinically as hemangioma or pyogenic granuloma and due to its clinical appearance an excisional biopsy was performed.

The histopathological features were similar to the first case and the final diagnosis was tufted angioma (Figures 2(c) and 2(d)). The patient continues to be clinically followed up, without recurrence after 6 months of follow-up (Figure 3(b)).

## 4. Discussion

The tufted angioma is a rare benign lesion, though its reactive or neoplastic nature is unclear. Some authors associate this disease with endocrine stimuli, such as the loads of hormonal release that occur at puberty suggesting that it may represent a reactive vascular proliferation secondary to hormonal stimulation [12]. However most previously reported cases of cutaneous TA are in infants or young children without gender predilection [11]. Notwithstanding, according to the literature, oral tufted angioma is most common in adults, where males are affected twice as much as females. In our first case reported, a history of trauma was identified, which corroborates with the reactional nature hypothesis for this lesion.

Most frequently, the cutaneous TA is clinically characterized as a solitary tumor with erythematous, macula, or plaque appearance located in the skin of neck, upper trunk, and extremities. Sometimes it can be associated with hyperhidrosis or hypertrichosis and Kasabach-Merritt syndrome (KMS), which is a rare coagulopathy resulting in thrombocytopenia, anemia, and hypofibrinogenemia^2^. However, the oral mucosa tufted angiomas reported so far, including the ones here reported, have not been associated with neither hyperhidrosis nor KMS [2, 13, 14].

The oral mucosal involvement by TA is extremely rare. These lesions usually are asymptomatic and display a papular or nodular aspect. They may grow rapidly and their color ranges from red-brown to purple-blue. Most of cases are localized in lip [1, 10, 11]. The characteristics observed in the present reported cases are similar to previous reports, in which the lesion observed is characterized as a firm, red to purple nodule.

Clinical differential diagnosis of the tufted angioma in lip should include reactive lesions, such as pyogenic granuloma and mucocele and benign neoplasms from endothelial or salivary gland origin. However, the histopathological findings are specific and these lesions exhibit individual characteristics by which they can be distinguished from TA [10].

The histopathology analysis of the herein reported cases revealed intense endothelial cell proliferation in nodular arrangements in a “cannon ball” fashion and numerous irregular capillary blood vessels with a slit-shaped lumen, consistent with TA diagnosis. Based on the histologic features it is important to distinguish TA from other vascular lesions such as pyogenic granuloma, haemangioma, and kaposiform hemangioendothelioma. The pyogenic granuloma shows a high vascular proliferation as capillaries and the presence of intense inflammatory infiltrate containing young fibroblasts that resemble granulation tissue, while tufted angioma is characterized by proliferation of spindled and polygonal cells surrounding vascular channels, organized as tufts [13]. The capillary haemangioma presents proliferation of endothelial cells forming lobular arrangement of well-formed capillaries; however, the clusters of endothelial cells in TA are larger and more irregular in shape. Moreover, the “cannon ball” distribution of nodules is only observed in TA [2]. The kaposiform hemangioendothelioma exhibits spindled endothelial cells with slit-like vessels and these cells grow in sheets or coalescing nodules rather than dispersed tufts as observed in TA [9, 13].

The treatment of TA is mostly performed by complete excision of the lesion. Furthermore, other methods were used previously by different authors, including compression therapy, surgery, cryotherapy, laser, topical or systemic corticosteroids, interferon, and chemotherapy [2, 9, 11]. On the other hand, some authors suggested no need of removal for skin TA, only monitoring the lesion, justifying that many lesions show spontaneous regression [9, 11]. However, this information is not applicable for oral TA, since all lesions reported have been removed, with exception of the case reported by Chaves et al. (2009) [1], where only an incisional biopsy was performed. Additionally, recurrence is common if the oral lesion is not completely removed [9, 14], as observed in both of the cases reported here. Thus, monitoring of patients with tufted angioma should be performed in order to prevent recurrences and complications especially in early childhood.

In conclusion, dentists should be aware of recurrent tufted angioma diagnosis in children and the reported cases may contribute to databases of the literature to provide a better understanding of the biological behavior and treatment of this lesion in children.

## Figures and Tables

**Table 1:** Tufted angioma's cases reported with oral mucosa involvement.

Case Report (year)	Sex	Age	Anatomic Location	Size	Duration	Treatment	Recurrence	Follow-up
Daley, 2000 [13]	F	20	Lower lip	0.1 cm diameter	10 years	Excision	NA	No
Kleinegger et al., 2000 [11]	M	34	Upper lip	0.3 cm diameter	NA	Excision	NA	No
Kleinegger et al., 2000 [11]	M	52	Anterior floor of the mouth	0.2 × 0.2 cm	10 days	Excision	No	3 months
Lee et al., 2006 [15]	F	36	Lower lip	0.5 × 0.8 cm	3 months	Excision	No	3 months
Chaves et al., 2009 [1]	M	12	Upper lip	0.8 cm diameter	2 months	Incisional biopsy	NA	NA
Sabharwal et al., 2013 [10]	M	47	Upper lip	0.5 × 0.5 cm	2 months	Excision	No	3 years
Sabharwal et al., 2013 [10]	M	40	Anterior dorsal tongue	0.5 × 0.5 cm	4 months	Excision	NA	NA
Present case 1	M	10	Labial commissure	1 cm diameter	2 months	Excision	Yes	1 year
Present case 2	M	12	Upper lip	1 cm diameter	4 months	Excision	Yes	6 months

W: Woman; M: Men; NA: Not available; No: Not realized.
